# Implementation and Knowledge of the Clinical Practice Guide for Palliative Care in the Ecuadorian Primary Care Level

**DOI:** 10.3390/ijerph182111573

**Published:** 2021-11-04

**Authors:** Tamara Rodríguez Quintana, Viviana Dávalos-Batallas, Ana-Magdalena Vargas-Martínez, Lucelly López, Patricia Bonilla-Sierra, María-de-las-Mercedes Lomas-Campos, Fatima Leon-Larios

**Affiliations:** 1 Department of Health Sciences, Universidad Técnica Particular de Loja (UTPL), Loja 110107, Ecuador; trodriguez3@utpl.edu.ec (T.R.Q.); vddavalos@utpl.edu.ec (V.D.-B.); pbonilla65@utpl.edu.ec (P.B.-S.); 2 Nursing Department, School of Nursing, Physiotherapy and Podiatry, University of Seville, 41009 Seville, Spain; mlomas@us.es (M.M.L.-C.); fatimaleon@us.es (F.L.-L.); 3 Research Department, School of Medicine, Universidad Pontificia Bolivariana, Calle 78B # 72A-109, Medellín 050001, Colombia; lucelly.lopez@upb.edu.co

**Keywords:** clinical practice guide, palliative care, knowledge

## Abstract

Ecuador assumed the commitment of including Palliative Care (PC) in its health policies. In 2014, the Ministry of Public Health (Ministerio de Salud Pública, MSP) approved the Clinical Practice Guide for Palliative Care (*Guía de Práctica Clínica sobre Cuidados Paliativos*, GPCCP), with application at the national level, as a mandatory internal regulation in all institutions belonging to the National Health System. In 2021, there is no evidence about the degree of implementation. The objective was to evaluate the implementation (I) of the GPCCP guide and the knowledge (C) of the health personnel working in the Zone 7 Health Centers (HCs). This is a cross-sectional, descriptive, and prospective study. A total of 292 professionals were interviewed: managers (38), physicians (150), and nurses (104). Three surveys based on the GPCCP guide were elaborated: one for the implementation, which was applied to the individuals in charge, and the others to assess the health professionals’ knowledge. The SPSS program was used, version 25. In the three groups, more than half of the participants had no training in PC, 91.2% of the HCs have the GPCCP guide, there is PC medical history (MH) in 38.2%, and morphine is used in 14.7%. The implementation of the GPCCP guide was inadequate in 52.9% of the cases. Only 25% treat the agony symptoms and 30%, delirium; 4.4% acknowledge the use of morphine in dyspnea, and 13.3% identify the subcutaneous route as the first choice for hydration at the end-of-life phase. Strategies to implement the GPCCP guide and to improve the health personnel’s knowledge must be implemented in Zone 7 centers.

## 1. Introduction

The view of palliative care (PC) in the world has changed over the years. It is acknowledged that it relieves suffering, controls symptoms, and improves quality of life and care quality [1]. Comprehensive care for patients with diseases that restrict life during their evolution offers an effective outcome–cost ratio for the patient, the family, and the health systems [2].

The Political Statement of the High-Level Meeting of the United Nations’ General Assembly on prevention and control of non-communicable diseases (2014) acknowledges the need to improve access to palliative care. The recommendation is to develop and implement palliative care policies to strengthen health systems [3]. Cost-effective and equitable palliative care services must be integrated in all levels, with an emphasis on primary, community, and home care [4].

The World Health Organization (WHO) proposes four key elements as a strategy to improve PC, namely: policy, education, availability of medication, and implementation of services [5]. The policy is considered indispensable to satisfy the other three elements; however, the existence of national PC laws or plans does not ensure that there will be more PC services, at least in Latin America (LA).

In Latin America, with a population of more than 600 million inhabitants, 75% of the population dies due to non-communicable diseases. In 2018, more than 3.5 million people living with some severe health problems were reported, reason why it is indispensable to continue developing this care. Despite this increase, the care provided is still insufficient and unequal [6,7]. Currently, coverage of PC needs in Latin America is only 7.6% and only 3.5% in Ecuador [8].

Important changes have been implemented in the public and health policies, favoring the integration of PC in the National Health System and assisting in the creation of public policies aimed at developing and implementing PC in Ecuador. In 2014, and as part of these strategies, the Ministry of Public Health approved the Clinical Practice Guide for Palliative Care and established its application in the entire National Health System as a mandatory internal regulation [9].

In relation to the training of future health professionals, although inclusion of the PC component in the Health Sciences courses is mandatory, previous studies have found that it is still necessary to advance in training, as a number of knowledge gaps have been identified [10,11]. 

The purpose of this article is to assess the implementation degree of the MSP Palliative Care Guide in Zone 7 Health Centers, as well as the level of knowledge about palliative care of the health personnel working in these centers, six years after approval of the Palliative Care National Plan in Ecuador

## 2. Materials and Methods

This was a cross-sectional, descriptive, and cross-sectional study. It was developed during the 2019–2020 period in Health Zone 7, which encompasses three provinces: Loja, El Oro, and Zamora Chinchipe. 

### 2.1. Population and Sample

There are 1554 health centers that depend on the Public Health Ministry (Ministerio de Salud Pública, MSP) in Ecuador. Health Zone 7, comprised by the provinces of Loja, El Oro, and Zamora Chinchipe, has 153 health centers, 66 of them in urban areas. The total population of this zone is 1,341,334 inhabitants who receive care, some in MSP health units and others in units from the public health network such as the different health centers belonging to the various social insurance systems in the country.

To assess the implementation of the GPCCP guide, the administrative managers of 38 health centers in urban areas where the Family and Community Medicine specialization students were doing their internships were interviewed. To assess the health personnel’s level of knowledge about palliative care, all the administrative personnel, physicians, and nurses who met the inclusion criteria were interviewed.

The following were established as inclusion criteria: for the health professionals in managerial positions, holding such positions for at least three months; and, for the physicians and nurses, working in the health center for at least three months; there were no exclusion criteria. Non-probabilistic convenience sampling was performed.

### 2.2. Ethical Considerations

All the participants were informed about the study objectives and were asked to sign a written informed consent of their participation in the study. They were guaranteed anonymity of the information provided and data confidentiality. Approval was obtained from the Committee of Ethics in Research with Human Beings of UTPL under Edict No. UTPL-CEISH-2020-RI05; in addition, authorization from the Zone 7 Health Coordination Office and the participants’ written and signed informed consent were also obtained. 

### 2.3. Procedures and Data Collection

To assess the implementation of the Palliative Care Guide, an ad hoc survey was applied, based on the guidelines set forth in the MSP guide, consisting of 27 questions: 11 collecting general information and the rest being specific about implementation of the guide. This instrument was applied to the health center administrative manager, and was self-administered with the interviewer present. From the specific questions, inadequate implementation was only considered when one of the answers was negative.

Whereas, to assess the knowledge about PC among the health personnel, separate surveys for the physicians and for the nursing personnel were elaborated. Both included 34 items, 9 for collecting general data of the interviewee and the rest to assess knowledge about palliative care. The two surveys were based on the guidelines set forth in Ecuador’s GPCCP guide, designed by the research authors and applied in the health centers.

### 2.4. Instrument

Information was collected about sociodemographic and labor variables, as well about experience and training in palliative care. Specifically, the variable related to the training hours, which was asked categorically, was transformed into a numerical variable for certain analyses that are detailed later in this article (“0 h” = 0; “less than 20 h” = 10; “between 20 and 50 h” = 35; “between 50 and 100 h” = 75; and “more than 100 h” = 100).

Questions were asked about the existence of the GPCCP guide, the annual training plan including the theme, number of training sessions, written performance protocol for coordination with the reference hospital, palliative care medical history, essential PC medications, population that required palliative care, trained interdisciplinary team, time available for the PC team to evaluate the first appointment of a patient with palliative needs in external consultation, coordination between the different assistance services to ensure care continuity, opioid availability, and other techniques that can help relieve the symptoms.

A number of variables were studied, such as: instruments used in PC, medication use, criteria to include patients in the program, number of symptoms that are assessed with the Edmonton scale, use of a prognostic scale to predict survival in a patient on PC, use of another scale to assess pain, criteria to include a patient with an advance-stage disease and a life expectancy of less than one year in the PC program, ICD-10 code used for palliative care, criteria to refer a patient, use of morphine at the end-of-life phase, first choice route for home hydration, aspects included in the psychosocial evaluation of the patient and the family, information provided by the family physician about the terminal phase and the need to transfer the patient to palliative care, about the changes in evolution of the disease, about palliative sedation and identification of pathological grief, among others.

### 2.5. Statistical Analyses 

In data description, absolute and percentage frequencies were used for the qualitative variables; and means and standard deviations were employed for the quantitative variables. In addition, a comparison of means and frequencies by professional group (managers, physicians, and nurses) was carried out, showing statistical significance after analyses by means of the t-test and ANOVA or Pearson’ chi-square tests, depending on the type of variable. Likewise, an analysis by means of Pearson’s chi-square was performed to compare the physicians’ and nurses’ knowledge about Palliative Care regarding certain aspects of the Clinical Practice Guide. The relationship between this knowledge and the years of general professional experience in palliative care and training in this area for each professional group (physicians and nurses) was studied using t-test, except for the variable related to first choice hydration route for patients at the end-of-life phase in their homes for which the ANOVA analysis was used. Data processing was performed in R statistical software (version R-4.0.5, Free Software Foundation’s GNU General Public License: https://www.r-project.org/about.html, access date: 18 May 2021). A 5% statistical significance level was adopted in all the analyses. 

## 3. Results

Descriptive characteristics of the sample are shown in Table 1. The total of subjects studied was 292, divided as follows: 38 health center administrative managers, 150 physicians, and 104 nurses.

In relation to the sociodemographic characteristics, women prevailed in the three groups, which represented more than half of the participants, reaching approximately 80% in the group of nurses. The overall mean age of the sample was around 37 years old. Among the administrative managers of the centers participating in the study, generalist physicians were a majority with 76.5%, and only 14.7% were specialists in Family and Community Medicine. Among the physicians, 38% were generalists, 28% were specialists in Family and Community Medicine, and 14.67% were surgeons.

The general professional experience presented a mean of 11 years, exceeding a mean of 12 years in the group of physicians. Less than 50% of the subjects interviewed acknowledged having experience in palliative care, and the nurses were those who most reported having had experience and whose mean time of experience in this care field was longer. 

Regarding the type of training received, medical professionals were those who most underwent graduate training and continuing education, whereas nursing professionals asserted having mostly undergone university undergraduate training. 34.5% of the administrative managers who stated having undergone training did so at the undergraduate level, 10.3% in continuing education and only 7% at the graduate level. In the case of the physicians, 33% state having undergone undergraduate training, 15.5% continuing education and only 9.3% graduate education, whereas 38.6% of the nurses underwent PC training at the undergraduate level, 13.6% in continuing education, and only 4% at the graduate level. In addition, 52.3% attended less than 20 h of training in this topic (Table 1).

### 3.1. Implementation of the Clinical Practice Guide for Palliative Care in the HCs

When analyzing the criteria to assess the implementation of the Clinical Practice Guide for Palliative Care based on the information provided by the administrative managers (Table 2), it was found that 91.18% of the health centers have this Guide; whereas there is a training program that includes PC only in 35.29% of the cases. There is a written performance protocol for coordination with the reference hospital in 36.36% of the HCs. It is noteworthy that there is PC medical history only in 38.24% of the cases. 

In relation to the essential PC medications that must be included in the basic medication chart in primary care units, it was found that there is Tramadol in 52.9% of the HCs and, regarding Morphine, it is present in only 14.7%. Other medications were also found in proportions below 50%, such as Amitriptyline (26.5%) and Haloperidol (21.2%). However, there is opioid availability in slightly more than 40% of the health centers.

The interdisciplinary teams for this type of care have physicians trained in PC (57.58%), with trained nurses (34.38%), a psychologist, and a social worker (18.75%) of the teams and only one physiotherapist (6.25%); whereas only 36.36% acknowledges that the regulated time available for the team to evaluate the first appointment is 60 min, according to the MSP’s PC care standard. Slightly more than half of the health centers coordinate care with other secondary care level hospital institutions 24 h a day, considering that the study area does not have tertiary-level hospitals or 24 h home care teams. 

The following were mentioned among the difficulties acknowledged to implement the MSP’s palliative care model flowchart, with integration of all the resources: lack of training in the theme, deficit in human resources and medications/medical inputs, limited time availability to provide care, and the physicians’ instability in contractual terms, including professionals who comply with the one-year rural service and others with fixed contracts.

### 3.2. Knowledge and Handling of the CPG Guide for PC by Physicians and Nurses

Approximately 50% of the physicians use both the ESAS and Pfeiffer’s and Karnofsky’s scales when approaching patients in palliative care. 24.83% uses some survival prediction scale; whereas 60.42% of the physicians interviewed use a scale to assess pain and more than one-third employ survival time and the symptoms as criteria to include a patient with a chronic disease in an advanced stage and lower life expectancy in the program. 

Nearly one-third of the physicians know the ICD-10 code corresponding to palliative care and 62.5% refer the patient to a PC unit when diagnosing an incurable disease and shortened life prognosis, always based on the disease, the life prognosis, and the expected evolution (Table 3). The end-of-life symptoms that are treated by almost all the physicians are severe pain, constipation, and vomiting. It draws the attention that only 20.91% treat agony symptoms and 25.89%, delirium. In relation to the use of morphine at the end-of-life phase, 76.3% use it for pain; however, only 5.41% acknowledge the need to use it in dyspnea and 27.12% state using it for palliative sedation although it is a non-hypnotic analgesic drug.

In relation to the first-choice route to hydrate patients at the end-of-life phase in their homes, 71.32% prefer the intravenous route and only 16.96% has a preference for the subcutaneous route, which is the one recognized and recommended in the CPG guide (Table 3). This is similar among nurses, referring to the intravenous route as the first choice for home rehydration. The differences between physicians and nurses regarding the proportions in their answers were statistically significant.

Regarding the knowledge investigated in physicians and nurses, it can be seen (Table 3 and Table 4) that there are no statistically significant differences in their answers, except for the assertions related to: “Morphine is the standard used to compare the analgesic effect of other opioids” and “People who take opioids should adopt certain measures to improve bowel elimination”, where the physicians showed greater knowledge.

When investigating how physicians assess the psychosocial sphere of the patients in palliative care and of the families, it was found that approximately 90% address the impact of the disease, although less than 70% evaluate the spiritual resources. More than 90% of the interviewees inform the family about the patient’s terminal phase and about the transfer to palliative care, as well as about evolution and palliative sedation. 67.59% is able to recognize pathological grief. However, around 25% of the physicians feel trained in the diagnosis and management of urgencies in PC and 55.78% feel trained to provide psychosocial and spiritual support to patients and families (Table 5). 

Regarding the nurses’ knowledge about different aspects included in the GCPCP guide, it is worth noting that 48% relates PC to deterioration or worsening of the clinical condition, which is associated with the fact that 73% consider that the pain treatment method is based on disease extension and not on its severity, and that only 53% of the interviewees consider that the PC philosophy is compatible with active treatments. In this line, they consider addiction as a problem for the use of morphine in patients who require long-term pain treatment in the PC context. Seventy-five percent of the nurses consider that it is appropriate to use a placebo in pain treatment. On the other hand, less than 50% acknowledge the impact of the disease and of fatigue on the manifestation of physical pain. More than 50% and 75% of the interviewees ignore the secondary effects of codeine and of meperidine, respectively.

Regarding the psychoemotional aspects and team care, it draws the attention that 60% believe that the accumulation of losses makes development of the Burnout syndrome inevitable in health personnel and that 34% did not answer this question. In relation to grief or loss, 72% consider that they are easier to solve when there is a distant or conflictive relationship. 

### 3.3. Relationship between General Knowledge about the Different Aspects of PC and the Physicians’ and Nurses’ Training and Experience

General professional experience showed a statistically significant difference in the group of physicians with the knowledge related to “Morphine is the standard used to compare the analgesic effect of other opioids”, “Adjuvant therapies are important in pain management”, and “The drugs that can cause respiratory depression are appropriate to treat severe dyspnea”. Among the nurses, a statistically significant relationship was only observed for the following assertion: “Meperidine is not an effective analgesic in the control of chronic pain” (Table 6).

Regarding the specific professional experience in PC, it only showed a statistically significant relationship among the nurses for the following assertion: “Somnolence associated with electrolyte imbalance can reduce the effect of sedation”, noticing more in-depth knowledge as the years of experience increase (Table 6).

Training in PC did not prove to be a determining factor in the improvement of knowledge about the different aspects contemplated in the GPCCP guide, both in physicians and in nurses. However, in most of the assertions, there is coincidence of a greater number of training hours with the fact of knowing these aspects, when compared to those who gave negative answers to these questions (Table 7).

## 4. Discussion

This study intended to analyze the impact of implementing the Clinical Practice Guide for Palliative Care in Ecuador’s Health Zone 7, since its approval in 2014. To such end, physicians and nurses involved in palliative care were asked about their knowledge and professional experience. Likewise, the managers of the health centers involved were also included.

Insufficient implementation of the CPCCP guide is evidenced, despite approval of the 2015–2017 Palliative Care National Plan, where all health establishments are compelled to apply it, which does not necessarily lead to putting it into practice to ensure provision of the service. This phenomenon has been observed in several studies [12,13] and, perhaps, the most notorious is the one by the WHO in 2015, which concludes that, in order to have successful Palliative Care programs, universal access to the essential PC medications is required, as well as generalized education and implementation, in line with the results found in this study. In addition, adequate funding and political commitment are required [10,14].

Nine out of 10 administrative managers stated that the demand for PC among the population of their health centers was less than or equal to 50 individuals, although chronic diseases in advanced stages are the most prevalent in Ecuador [15]. This can be due to the fact that they are not properly identified or that they are only considered at the end of the chronic process, instead of at early stages, as currently recommended [16]. 

Clinical practice guides are important for decision-making, especially in primary care [17,18]; however, their insufficient implementation precludes timely care to the patients with progressive diseases at advanced stages, thus contributing to quality-of-life deterioration and to increased suffering [1]. Usually, one of the main causes of insufficient implementation is lack of knowledge about their existence [10,19]; however, not only their knowledge by the health professionals involved must be weighted, but also receiving training on how to implement them and that access to the necessary resources is ensured. The fact that the participants possess little training in PC is an expected result, as other authors have acknowledged that, in the Ecuadorian context, training is still limited, both at the undergraduate and graduate levels, thus translating into low identification of individuals in need of this type of care and highlighting the need to maintain continuing education [10,11,20]. It is necessary to develop a specific training program on palliative care, both at the undergraduate and graduate levels for nurses and physicians, in order to optimize the care provided [21]. Even if Ecuador has recognized the palliative care medical specialty, it is necessary to keep advancing in the development of homogeneous contents that ensure quality care [20]. Not all the health professionals involved in palliative care who participated in the study had undergone training or had the medical specialty. Certain labor instability in these professionals to be able to do their job should be added to the aforementioned, since stability is sometimes hindered because they need to change work centers many times in a brief period of time [10]. Nevertheless, it was indeed noticed that those individuals who had received previous training knew more aspects of the guide and concepts related to palliative care, a reason why the knowledge acquired exerts a positive impact on palliative care management [22,23].

It is worrying that health personnel have insufficient knowledge about PC. In the literature that was consulted, the importance of this personnel possessing adequate knowledge to improve quality of life and reduce anguish has been highlighted [19]. Few of them treat agony, dyspnea, and delirium, being necessary to know correct management of the opioids that help alleviate suffering [24,25]; for them, training should be addressed that removes the myths and erroneous concepts around the use of these substances, such as those related to addiction to morphine derived from its use [26]. During the 67th World Health Assembly, the WHO urged the countries to ensure funding and allocate resources that include availability of essential medications for symptom relief [10]. It is necessary to sustain advances in the provision of resources and in the involvement of governments in policies that ensure palliative care.

Regarding the psychosocial approach, it was noticed in the study that the psychological and social sphere were taken into account, but to the detriment of the spiritual, despite the benefits associated with quality of life when the spiritual sphere is included [27,28]. The professionals felt less prepared for the spiritual approach to the patient and the family [29], as well as for diagnoses and urgencies in palliative care [30]. Therefore, the spiritual sphere should be taken into account and strategies should be implemented to improve coping in health professionals.

### Limitations

The authors consider that this study allows having an idea of how the implementation process of the palliative care guide was carried out, although it is not to be forgotten that it was conducted in a single Ecuadorian zone, which is why the results cannot be generalized to the entire country. In addition, it should be analyzed if the situation is even worse in the country’s rural and remote areas, where health services are even scarcer. As this is a cross-sectional study, no causality of what is detailed can be established. However, this study focuses attention on the need to know the degree of implementation of the palliative care guide in other health areas, since it is necessary to keep researching policies and strategies that turn out to be successful so that the implementation of palliative care attains high standards in the country.

## 5. Conclusions

The implementation of the GPCCP guide in Zone 7 Health Centers is insufficient. Among the main difficulties encountered are lack of information about the guide, specific training on its application, and limited availability of essential PC medications and opioids. The health professionals possess insufficient knowledge about PC and limited professional experience. They acknowledge that they do not feel trained in the diagnosis and management of PC urgencies, in the use of morphine for pain treatment, and in its secondary effects. It becomes necessary to keep working on the training of the health professionals involved in palliative care, improving their care skills, especially in the spiritual sphere, as well as to enhance accessibility to the drugs which are required for an effective approach. It would be desirable to reproduce this work in other areas of the country, to know the degree of implementation of the guide at the national level, and improve the degree of knowledge and training about palliative care. It seems necessary to include training in palliative care in undergraduate degree programs in the health sciences, as well as in postgraduate education.

## Figures and Tables

**Table 1:** Characteristics of the sample (n = 292).

Variables	Total	Managers (n = 38)	Nurses (n = 104)	Physicians (n = 150)
***Sociodemographic***				
**Gender [female]**	199 (69.1)	19 (55.9)	83 (79.8)	97 (64.7) ***
** $\mathrm{Age}\;\bar{x}$ ** **(SD)**	37.49 (10.80)	35.32 (9.52)	37.38 (10.50)	38.05 (11.27)
**Canton**				
Loja	20 (52.63)	20 (52.6)	N.A.	N.A.
Machala	3 (7.89)	3 (7.89)	N.A.	N.A.
Pasaje	1 (2.63)	1 (2.63)	N.A.	N.A.
Yacuambi	2 (5.26)	2 (5.26)	N.A.	N.A.
Yantzaza	2 (5.26)	2 (5.26)	N.A.	N.A.
Zamora	5 (13.16)	5 (13.16)	N.A.	N.A.
Zaruma	5 (13.16)	5 (13.16)	N.A.	N.A.
**Cycle**				
I	13 (6.19)	13 (39.4)	0 (0.0)	0 (0.0) ***
IV	11 (5.24)	2 (6.1)	3 (4.3)	6 (5.6)
V	9 (4.29)	1 (3.0)	4 (5.8)	4 (3.7)
VI	120 (57.14)	8 (24.2)	43 (62.3)	69 (63.9)
IX	57 (27.14)	9 (27.3)	19 (27.5)	29 (26.9)
**Parrish**				
Catamayo	35 (13.89)	1 (2.6)	9 (11.0)	25 (18.9) ***
Machala	56 (22.22)	1 (2.6)	25 (30.5)	30 (22.7)
Pasaje	18 (7.14)	1 (2.6)	7 (8.5)	10 (7.6)
Zamora	53 (21.04)	3 (7.9)	19 (23.2)	31 (23.5)
Others	130 (35.71)	32 (84.2)	22 (26.8)	36 (27.3)
***Professional training and experience***				
**$\mathrm{Professional}\;\mathrm{experience}\;(\mathrm{years})\;\bar{x}$** **(SD)**	11.08 (9.54)	9.73 (8.61)	10.19 (8.53)	12.02 (10.33)
**$\mathrm{Time}\;\mathrm{as}\;\mathrm{administrative}\;\mathrm{manager}\;(\mathrm{years})\;\bar{x}$** **(SD)**	3.34 (7.24)	3.34 (7.24)	NA	NA
**Experience in PC [Yes]**	116 (40.28)	13 (38.2)	47 (45.2)	56 (37.3)
**Experience in PC (years)**	1.89 (3.74)	1.90 (4.39)	2.54 (4.91)	1.46 (2.43)
**Training in PC (hours)**				
0 h	17 (8.72)	2 (7.7)	0 (0.0)	15 (16.0) ***
Less than 20 h	102 (52.31)	14 (53.8)	50 (66.7)	38 (40.4)
Between 20 and 50 h	53 (27.18)	7 (26.9)	17 (22.7)	29 (30.9)
Between 50 and 100 h	13 (6.67)	1 (3.8)	4 (5.3)	8 (8.5)
More than 100 h	10 (5.13)	2 (7.7)	4 (5.3)	4 (4.3)
**Graduate Courses in PC [Yes]**	15 (7.11)	2 (7.1)	3 (4.0)	10 (9.3)
**Higher Education in PC [Yes]**	149 (65.07)	10 (34.5)	32 (38.6)	38 (32.5)
**Continuing Education in PC [Yes]**	31 (14.09)	3 (10.3)	11 (13.6)	17 (15.5)
**Higher Education in PC + Continuing Education in PC [Yes]**	15 (7.32)	4 (15.4)	8 (10.7)	3 (2.9) **

*Note:* $\bar{x}$ (SD), the mean values and standard deviation are shown in between round brackets when the variable is numerical. The frequency and percentage are shown in between round brackets when the variable is categorical. *** and ** represent 1% and 5% statistically significant differences between the variables by professional category (administrative clerk, nurse and physician). N.A. (Not applicable).

**Table 2:** Implementation of the Clinical Practice Guide for Palliative Care.

	N	%
CPG for Palliative Care [Yes]	31	91.18
Annual training program in the HC (including PC) [Yes]	12	35.29
Number of annual training sessions	1 (SD: 1.98)	
Coordination protocol with reference hospital [Yes]	12	36.36
Medical History in PC [Yes]	13	38.24
Availability of essential PC medications suggested by the WHO [Yes]		
Paracetamol/Ibuprofeno/Diclofenac/Ketorolac/Naproxen	33	97.06
Codeine/Tramadol	18	52.94
Morphine/Buprenorphine	5	14.71
Butylscopolamine/Hyoscine Bromide	24	72.73
Lactulose/Glycerol	25	73.53
Loperamide	1	3.03
Metroclopramide	32	94.12
Furosemide/Spironolactone	27	81.82
Omeprazole	33	97.06
Metronidazole	34	100
Prednisone/Dexamethasone	26	78.79
Fluconazole	34	100
Fenitoine/Phenobarbital/Clonazepam/Carbamazepine/Valproic Acid/Alprazolam/Diazepam	27	79.41
Amitriptyline	9	26.47
Haloperidol	7	21.21
Salbutamol	32	94.12
Opioid availability [Yes]	14	41.18
HC population that required PC (2018)		
Less than 50	31	93.94
Between 50 and 100	1	3.03
Between 100 and 150	1	3.03
More than 150	0	0.00
Interdisciplinary PC team		
Physician	19	57.58
Nurse	11	34.38
Psychologist	6	18.75
Physiotherapist	2	6.25
Social Worker	6	18.75
Care time available, first appointment in external consultation		
15 min	1	3.03
30 min	13	39.39
45 min	7	21.21
60 min	12	36.36
Coordination between different 24 h care services [Yes]	17	51.52
Other techniques to be considered		
Hypnosis	4	12.50
Relaxation therapy	17	53.12
Aromatherapy	1	3.12
Homeopathy	1	3.12
Acupuncture	7	21.88
Ozone therapy	1	3.12
Reiki	1	3.12

**Table 3:** Use and knowledge about instruments, inclusion criteria, and prescription in PC by the physicians.

	Physicians				
	n	%		n	%
*Instruments*					
**ESAS** [knows it]	19	30.16	**Barthel** [knows it]	27	30.00
[Uses it]	31	49.21	[Uses it]	30	33.33
[Knows it and uses it]	13	20.63	[Knows it and uses it]	33	36.67
**NECPAL** [knows it]	24	35.82	**Symptoms assessed by the Edmonton scale**		
[Uses it]	31	46.27	0–3	28	22.95
[Knows it and uses it]	12	17.91	4–6	60	49.18
**Karnofsky** [knows it]	46	42.59	7–10	34	27.87
[Uses it]	22	20.37	**Use of the survival prognosis scale** [Yes]	36	24.83
			[No]	52	35.86
[Knows it and uses it]	40	37.04	[Does not know any scale]	57	39.31
**Pfeiffer** [knows it]	18	26.87	**Use of a scale to assess pain** [Yes]	87	60.42
			[No]	35	24.31
[Uses it]	33	49.25	[Does not know any scale]	22	15.28
[Knows it and uses it]	16	23.88			
*Inclusion criteria*					
**Patient with advanced-stage disease and life expectancy of less than one year** [it does include it]	124	83.78	**When a patient is referred to a PC unit**		
**Criterion/Criteria to include it**			When diagnosing an incurable disease and shortened life prognosis, always based on the disease, the life prognosis and the evolution expected	90	62.50
Time	15	10.87	The patient and/or family requires so	6	4.17
Presence of symptoms	11	7.97	Progressive and irreversible deterioration, with increase in the number of complications and/or needs	42	29.17
**Time and presence of symptoms**	112	81.16	In agony	3	2.08
**ICD-10 code**			Never	3	2.08
Z21	11	8.27	**Difficulty referring a patient to a PC unit**		
Z50.4	10	7.52	Yes, I don’t know any unit nearby	50	34.72
Z51.5	96	72.18	Yes, the family gets scared at the word “palliative”	11	7.64
Z52.1	16	12.03	Yes, I’d rather be treated in PHC	11	7.64
			No, I refer the patient so that they can be followed-up in both services (PHC and Hospital)	61	42.36
			No, I refer the patient so that they are followed-up in a PC unit	11	7.64
*Drug prescription*					
**Dyspnea**	51	44.35	**Agony**	23	20.91
**Delirium**	29	25.89	**Use of morphine as end-of-life treatment for:**		
**Vomiting**	74	63.79	Pain	103	76.3
**Constipation**	80	67.23	Palliative sedation	32	27.12
**Severe pain**	81	66.39	Dyspnea	6	5.41
**Asthenia-Anorexia-Cachexia**	38	32.48	Pain and dyspnea	37	31.36

**Table 4:** Comparison between physicians and nurses regarding knowledge/beliefs related to palliative care.

	Physicians		Nurses		*p*-Value
	**n**	**%**	**N**	**%**	
*Knowledge/Beliefs*					
**First choice hydration route for patients at the end-of-life phase in their homes**					
Oral	37	31.09	15	14.4	**0.003**
Subcutaneous	19	16.96	3	2.9	**0.001**
Intravenous	92	71.32	86	82.7	**0.003**
**Morphine is the standard used to compare the analgesic effect of other opioids** [Yes]	101	68.71	51	49.0	**0.002**
**Adjuvant therapies are important in pain management** [Yes]	132	89.80	90	87.4	0.551
**Somnolence associated with electrolyte imbalance can reduce the effect of sedation** [Yes]	73	49.66	52	50.5	0.898
**People who take opioids should adopt certain measures to improve bowel elimination** [Yes]	116	78.91	60	58.3	**0.001**
**The drugs that can cause respiratory depression are appropriate to treat severe dyspnea** [Yes]	32	21.77	15	14.6	0.154
**At high doses, codeine causes more nausea and vomiting than morphine** [Yes]	68	46.26	48	47.1	0.901
**Dolantine is not an effective analgesic in the control of chronic pain** [Yes]	29	19.73	25	24.5	0.369

*Note:* Pearson’ chi-square tests were used to compare the different variables related to knowledges/beliefs between doctors and nurses. Numbers in bold indicates that *p*-value is less than 0.05, being statistically significant.

**Table 5:** Management of the psychosocial sphere by physicians.

	N	%
Training to provide psychosocial and spiritual support to the patient and the family [Yes]	82	55.78
Training to diagnose and manage urgencies in PC [Yes]	37	25.17
*Psychosocial assessment (aspects included)*		
Impact of the disease [Yes]	131	89.73
Coping styles [Yes]	116	81.12
Spiritual resources [Yes]	97	68.31
*Information provided to the family*		
About the terminal phase and the need to refer to PC	139	94.56
About the changes in evolution of the disease	142	96.6
About palliative sedation	133	91.10
*Identification of pathological grief*		
Clinical case about pathological grief (situation that this person is going through)		
Depression	43	29.66
Anxiety	4	2.76
Pathological grief	98	67.59

**Table 6:** Physicians’ and nurses’ knowledge according to general professional experience and experience in palliative care.

	Professional Experience (Years)				Experience in Palliative Care (Years)			
	Medicine Mean (SD)	*p*-Value	Nursing Mean (SD)	*p*-Value	Medicine Mean (SD)	*p*-Value	Nursing Mean (SD)	*p*-Value
Morphine is the standard used to compare the analgesic effect of other opioids		**0.016**		0.466		0.368		0.171
*Yes* *No* *Prefers not to answer*	11.67 (10.22) 5.93 (4.86) 15.42 (11.26)		9.69 (7.79) 9.22 (7.98) 11.80 (10.09)		1.63 (2.45) 0.58 (0.79) 1.38 (2.81)		2.96 (5.07) 3.43 (6.62) 0.91 (1.08)	
Adjuvant therapies are important in pain management		**0.005**		0.300		0.861		0.580
*Yes* *No* *Prefers not to answer*	11.11 (9.77) 12.00 (0.00) 20.43 (12.58)		9.77 (8.06) 7.00 (0.00) 13.75 (11.82)		1.52 (2.54) 1.00 (0.00) 1.15 (1.34)		2.41 (4.25) 0.00 (0.00) 1.20 (1.87)	
Somnolence associated with electrolyte imbalance can reduce the effect of sedation		0.851		0.311		0.638		**0.021**
*Yes* *No* *Prefers not to answer*	11.96 (10.64) 11.41 (10.09) 12.79 (10.31)		10.35 (8.00) 8.33 (6.86) 12.00 (11.10)		1.68 (2.79) 1.24 (2.26) 1.32 (1.76)		3.34 (5.07) 1.30 (2.27) 0.68 (1.00)	
People who take opioids should adopt certain measures to improve bowel elimination		0.129		0.518		0.430		0.661
*Yes* *No* *Prefers not to answer*	11.39 (10.24) 10.00 (7.70) 16.05 (11.36)		10.28 (8.60) 7.93 (6.15) 11.14 (9.53)		1.34 (2.26) 1.89 (3.79) 2.05 (2.67)		2.56 (4.89) 1.50 (1.29) 2.04 (3.12)	
The drugs that can cause respiratory depression are appropriate to treat severe dyspnea		**0.006**		0.437		0.422		0.246
*Yes* *No* *Prefers not to answer*	7.38 (6.12) 14.25 (11.34) 11.49 (10.03)		9.10 (6.91) 9.31 (8.27) 11.58 (9.47)		0.97 (1.66) 1.57 (2.21) 1.72 (3.26)		1.46 (2.85) 3.02 (5.29) 1.63 (2.28)	
At high doses, codeine causes more nausea and vomiting than morphine		0.943		0.515		0.346		0.194
*Yes* *No* *Prefers not to answer*	12.31 (9.55) 11.58 (11.06) 11.83 (11.16)		9.39 (7.75) 9.93 (8.10) 11.50 (9.66)		1.75 (3.10) 1.61 (1.73) 1.08 (1.67)		3.02 (5.07) 1.23 (2.77) 1.57 (2.33)	
Dolantine is not an effective analgesic in the control of chronic pain		0.650		**0.028**		0.225		0.599
*Yes* *No* *Prefers not to answer*	11.75 (9.82) 10.71 (9.73) 12.64 (10.85)		7.80 (6.63) 6.38 (6.29) 11.92 (9.30)		1.32 (1.77) 0.91 (1.28) 1.78 (2.93)		1.95 (2.94) 1.38 (1.94) 2.57 (4.76)	

*Note*: *p*-value refers to the association studied between professional experience measured in years and the rest of the variables described in the first column of the table, as well as between the experience in palliative care and the rest of the variables, for each healthcare professional (doctors and nurses) separately. The statistical analysis used was an ANOVA analysis. Numbers in bold indicates that *p*-value is less than 0.05, being statistically significant.

**Table 7:** Physicians’ and nurses’ knowledge according to hours of training in palliative care.

	Training in Palliative Care (Years)			
	Medicine Mean (SD)	*p*-Value	Nursing Mean (SD)	*p*-Value
Morphine is the standard used to compare the analgesic effect of other opioids				
*Yes* *No* *Prefer not to answer*	27.21 (27.57) 20.83 (15.94) 21.00 (23.37)	0.588	26.25 (23.16) 18.25 (16.80) 25.53 (33.62)	0.491
Adjuvant therapies are important in pain management				
*Yes* *No* *Prefer not to answer*	27.08 (26.83) 35.00 (0.00) 9.44 (10.74)	0.146	25.55 (26.19) 35.00 (0.00) 12.78 (8.33)	0.325
Somnolence associated with electrolyte imbalance can reduce the effect of sedation				
*Yes* *No* *Prefer not to answer*	25.91 (26.79) 22.41 (22.70) 28.81 (29.58)	0.690	25.24 (26.85) 26.94 (21.22) 17.67 (23.67)	0.522
People who take opioids should adopt certain measures to improve bowel elimination				
*Yes* *No* *Prefer not to answer*	27.30 (27.53) 22.50 (13.69) 17.14 (21.10)	0.398	25.63 (28.58) 20.45 (20.67) 23.26 (19.98)	0.818
The drugs that can cause respiratory depression are appropriate to treat severe dyspnea				
*Yes* *No* *Prefer not to answer*	29.00 (29.89) 27.50 (24.15) 19.64 (26.31)	0.364	29.17 (34.43) 20.00 (15.28) 26.96 (29.39)	0.414
At high doses, codeine causes more nausea and vomiting than morphine				
*Yes* *No* *Prefer not to answer*	30.35 (26.65) 27.81 (27.32) 18.43 (23.91)	0.123	26.94 (26.76) 13.85 (9.39) 26.04 (27.27)	0.250
Dolantine is not an effective analgesic in the control of chronic pain				
*Yes* *No* *Prefer not to answer*	25.88 (26.53) 24.75 (21.24) 25.61 (27.86)	0.989	16.39 (16.70) 30.00 (18.59) 25.47 (28.84)	0.290

Note: *p*-value refers to the association studied between training in palliative care measured in years and the rest of the variables described in the first column of the table for each healthcare professional (doctors and nurses) separately. The statistical analysis used was an ANOVA analysis.

## Data Availability

The data that support the findings of this study are available from the corresponding author (A.-M.V.-M.) upon reasonable request.
